# Molecular chirality detection using plasmonic and dielectric nanoparticles

**DOI:** 10.1515/nanoph-2021-0649

**Published:** 2022-01-11

**Authors:** TaeHyung Kim, Q-Han Park

**Affiliations:** Department of Physics, Korea University, Seoul 02841, Republic of Korea

**Keywords:** chiral molecules, chiral sensing, chiroptical spectroscopy, circular dichroism (CD), dielectric nanoparticles, plasmonic nanoparticles

## Abstract

Nanoscale particles and structures hold promise in circular dichroism (CD) spectroscopy for overcoming the weakness of molecular CD signals. Significant effort have been made to characterize nanophotonic CD enhancement and find efficient ways to boost molecular chirality, but the best solution is yet to be found. In this paper, we present a rigorous analytic study of the nanophotonic CD enhancement of typical nanoparticles. We consider metallic and dielectric nanoparticles capped with chiral molecules and analyze the effect of multipolar nanoparticles on the molecular CD. We identify the spectral features of the molecular CD resulting from the electric and magnetic resonances of nanoparticles and suggest better ways to boost molecular chirality. We also clarify the contribution of particle scattering and absorption to the molecular CD and the dependence on particle size. Our work provides an exact analytic approach to nanophotonic CD enhancement and offers a rule for selecting the most efficient particle for sensitive molecular chirality detection.

## 1 Introduction

Nanoparticles and nanostructures are a promising new direction for circular dichroism (CD) spectroscopy. They can generate locally enhanced electromagnetic fields, which are necessary for sensitive molecular CD measurement. Various approaches have been tried to utilize nanostructures for CD spectroscopy, such as chiral metasurfaces [1], [2], [3], [4], [5], achiral nanoarrays [6], [7], [8], [9], [10], [11], achiral plasmonic nanoparticles [12], [13], [14], [15], [16], [17], [18], [19], [20], [21], and dielectric nanoparticles [12, 14, 15, 22, 23]. In these studies, CD enhancement was usually achieved near the resonance frequency of the nanostructures. The most studied resonant nanostructures, both theoretically [12], [13], [14], [15, 24] and experimentally [16], [17], [18], [19], [20], [21], [22], are achiral nanoparticles, whether plasmonic or dielectric. The main advantage of using achiral nanoparticles is separating the molecular CD signals from the unwanted strong background CD signals occurring in chiral nanostructures. In addition, achiral nanoparticles in the form of colloidal solutions do not require complex measurements while, for example, nanoarrays coupled with chiral molecules require additional experimental processes to obtain measurable CD signals [1, 7, 9, 10]. Despite the growing interest in achiral nanoparticles, theoretical studies on the effect of achiral nanoparticles on chiral molecules have mostly continued to apply conventional dipole approximation [12], [13], [14]. Although exceptionally some studies report chiroptical responses by higher-order multipoles [23, 24], these works do not consider the scattering-induced CD of a nanoparticle which results from nearby chiral molecules. Large-sized achiral nanoparticles capped with chiral molecules can be synthesized experimentally [16, 17, 19, 20] and offer better efficiency for CD enhancement through an enlarged surface area. However, a non-static analytic approach has remained unexplored.

In this paper, we investigate achiral nanoparticles capped with chiral molecules and obtain exact Mie scattering solutions. We identify the multipole resonances of both plasmonic and dielectric nanoparticles and their effect on the surrounding chiral molecules. The spectral features of CD enhancement caused by electric and magnetic resonances, are presented in detail. We find that, although plasmonic nanoparticles show only electric resonances, high index dielectric nanoparticles possess magnetic resonances which can boost the molecular CD more efficiently than electric resonances. By solving Mie scattering analytically, we also clarify the contribution of nanoparticle scattering and absorption to the molecular CD and their dependence on particle size. We also show that scattering by larger nanoparticles can significantly enhance the molecular CD. In particular, the scattering of a high-index nanoparticle combined with chiral molecules contributes critically to the overall higher-order induced CD. Consequently, we find that the higher-order resonances of large-sized achiral nanoparticles, especially magnetic resonance, are more effective for enhancing weak molecular CD signals. Our analytic approach based on Mie theory can provide both a qualitative understanding of and a good approximation to other non-spherical nanostructures for which analytic solutions are possible and is applicable to the development of more sensitive molecular chirality detection methods.

## 2 Chiral Mie theory for achiral core-chiral shell nanoparticle

**Figure 1:**
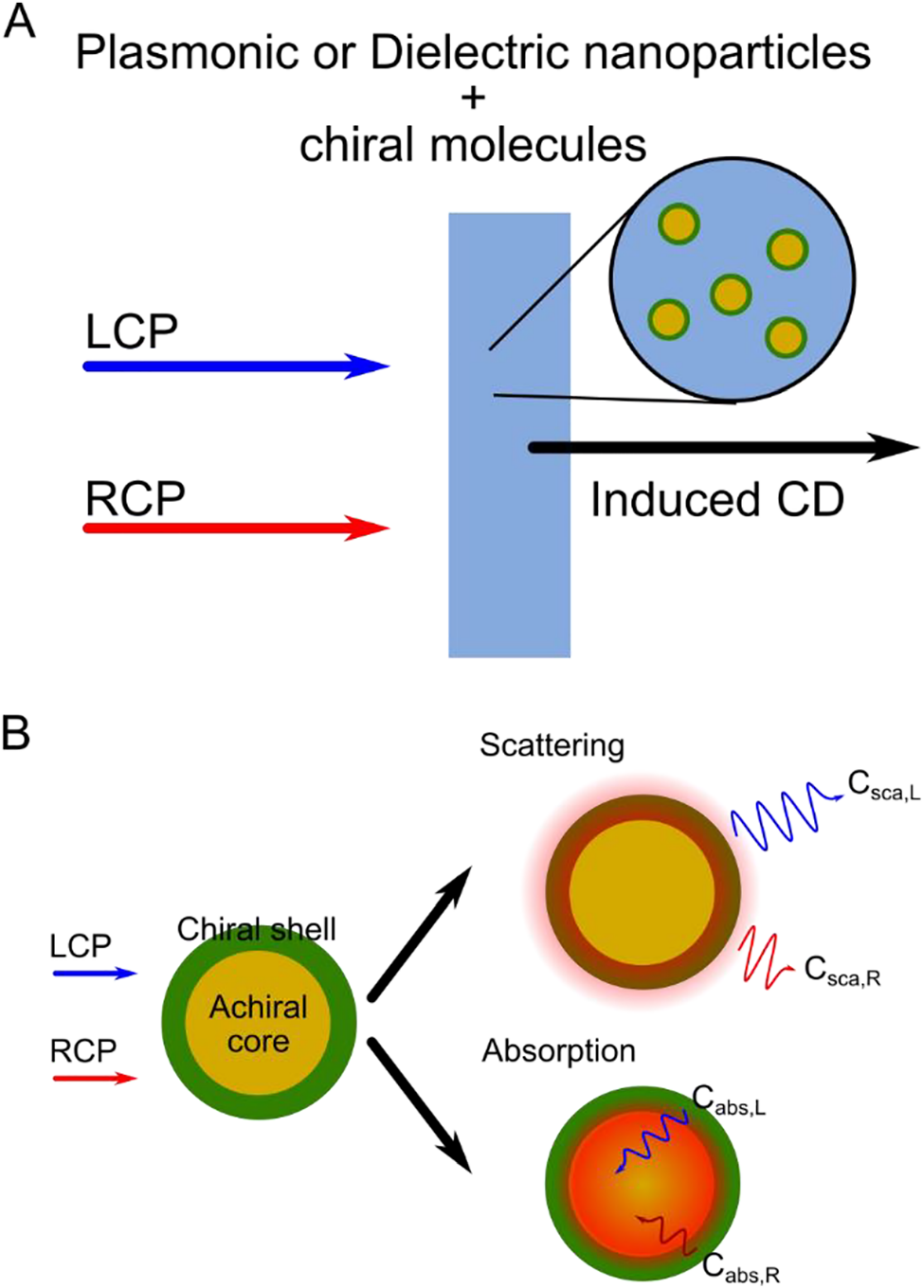
Schematic figure of CD measurement for (A) achiral plasmonic and dielectric nanoparticles capped with chiral molecules (B) schematic figure of Chiral Mie theory for large-sized achiral core-chiral shell nanospheres embedded in achiral background.

To understand analytically the chiroptical activity of chiral molecules, we consider a core–shell system with spherical core nanoparticles and a shell made of chiral molecules, as shown in Figure 1, and solve the associated Mie scattering problem. Firstly, we introduce the constitutive relation of a chiral medium D=ϵE+iκH and B=μH−iκE where ϵ , μ and κ are the permittivity, the permeability and the chirality parameter of a chiral medium, respectively [25]. To solve the Mie problem for a chiral medium, we express the incident, scattered and internal fields of our system in terms of $\left\{Q_{L}\left(k_{L}\right),Q_{R}\left(k_{R}\right)\right\}$ by using the wave-field decomposition method. In the chiral medium, the wavenumber of two circularly polarized lights of opposite handedness is given by $k_{L,R}=\left(n_{c}\pm \kappa \right)k_{0}$ with the refractive index $n_{c}$ and vacuum wavenumber $k_{0}=2\pi /\lambda$ . Plus and minus signs correspond to left and right circularly polarized light wavenumbers $k_{L}$ and $k_{R}$ , respectively. Using the vector spherical harmonics M and N of the Mie theory, we express the incident field by (see SI)

(1) $$Q_{L}^{i}=\sum_{n=0}^{\infty }Q_{L}i^{n}\frac{2n+1}{n\left(n+1\right)}\left[\left(M_{o1n}^{\left(1\right)}\left(k_{b}\right)+N_{o1n}^{\left(1\right)}\left(k_{b}\right)\right)-i\left(M_{e1n}^{\left(1\right)}\left(k_{b}\right)+N_{e1n}^{\left(1\right)}\left(k_{b}\right)\right)\right],$$

(2) $$Q_{R}^{s}=\sum_{n=0}^{\infty }Q_{R}i^{n}\frac{2n+1}{n\left(n+1\right)}\left[c_{n}\left(M_{o1n}^{\left(3\right)}\left(k_{b}\right)-N_{o1n}^{\left(3\right)}\left(k_{b}\right)\right)+id_{n}\left(M_{e1n}^{\left(3\right)}\left(k_{b}\right)-N_{e1n}^{\left(3\right)}\left(k_{b}\right)\right)\right],$$

and the scattered field by

(3) $$Q_{L}^{s}=\sum_{n=0}^{\infty }i^{n}\frac{2n+1}{n\left(n+1\right)}\left[\left(Q_{L}\alpha_{n}+Q_{R}\beta_{n}\right)\left(M_{o1n}^{\left(3\right)}\left(k_{b}\right)+N_{o1n}^{\left(3\right)}\left(k_{b}\right)\right)-i\left(Q_{L}\alpha_{n}-Q_{R}\beta_{n}\right)\left(M_{e1n}^{\left(3\right)}\left(k_{b}\right)+N_{e1n}^{\left(3\right)}\left(k_{b}\right)\right)\right],$$

(4) $$Q_{R}^{s}=\sum_{n=0}^{\infty }i^{n}\frac{2n+1}{n\left(n+1\right)}\left[\left(Q_{L}\gamma_{n}+Q_{R}\delta_{n}\right)\left(M_{o1n}^{\left(3\right)}\left(k_{b}\right)-N_{o1n}^{\left(3\right)}\left(k_{b}\right)\right)+i\left(-Q_{L}\gamma_{n}+Q_{R}\delta_{n}\right)\left(M_{e1n}^{\left(3\right)}\left(k_{b}\right)-N_{e1n}^{\left(3\right)}\left(k_{b}\right)\right)\right],$$

where $\alpha_{n}$ , $\beta_{n}$ , $\gamma_{n}$ , and $\delta_{n}$ are scattering coefficients, $Q_{L,R}$ is the amplitude of the left- and right-circularly polarized components of an incident plane wave and $k_{b}=n_{b}k_{0}$ is the wavenumber of the achiral background surrounding the core–shell system with the refractive index $n_{b}$ .

We solved the chiral Mie theory explicitly and calculated the rate of extinction, scattered, and absorbed energies normalized by the incident light energy (detailed derivations are given in SI). The resulting extinction, scattering and absorption cross sections are expressed by

(5) CextNP@mol=−4π∑n=1∞(2n+1)[QL2QL2+QR2Reαnkb2+QR2QL2+QR2Reδnkb2],

(6) $$C_{sca}^{NP@mol}=4\pi \sum_{n=1}^{\infty }\left(2n+1\right)\left[\frac{{Q_{L}}^{2}}{{Q_{L}}^{2}+{Q_{R}}^{2}}\frac{{\left|\alpha_{n}\right|}^{2}+{\left|\gamma_{n}\right|}^{2}}{{k_{b}}^{2}}+\frac{{Q_{R}}^{2}}{{Q_{L}}^{2}+{Q_{R}}^{2}}\frac{{\left|\beta_{n}\right|}^{2}+{\left|\delta_{n}\right|}^{2}}{{k_{b}}^{2}}\right],$$

(7) $$C_{abs}^{NP@mol}=C_{ext}^{NP@mol}-C_{sca}^{NP@mol},$$

where the scattering coefficients are determined from the boundary condition and n accounts for the order of resonance. We define the circular differential extinction cross section as the difference of extinction cross section for two opposite circularly polarized incident lights and hold the same definition for scattering and absorption. Then, the calculated circular differential extinction, scattering and absorption cross sections are

(8) ΔCextNP@mol=−4π∑n=1∞(2n+1)[Reαn−Reδnkb2],

(9) $$\Delta C_{sca}^{NP@mol}=4\pi \sum_{n=1}^{\infty }\left(2n+1\right)\left[\frac{{\left|\alpha_{n}\right|}^{2}-{\left|\beta_{n}\right|}^{2}+{\left|\gamma_{n}\right|}^{2}-{\left|\delta_{n}\right|}^{2}}{{k_{b}}^{2}}\right],$$

(10) $$\Delta C_{abs}^{NP@mol}=C_{abs}^{NP@mol}\left(Q_{L}=1,Q_{R}=0\right)-C_{abs}^{NP@mol}\left(Q_{L}=0,Q_{R}=1\right),$$

where the polarization states of the incident light are used with $\left(Q_{L},Q_{R}\right)=\left(1,0\right)$ and $\left(Q_{L},Q_{R}\right)=\left(0,1\right)$ for the left- and right-circular polarization respectively.

**Figure 2:**
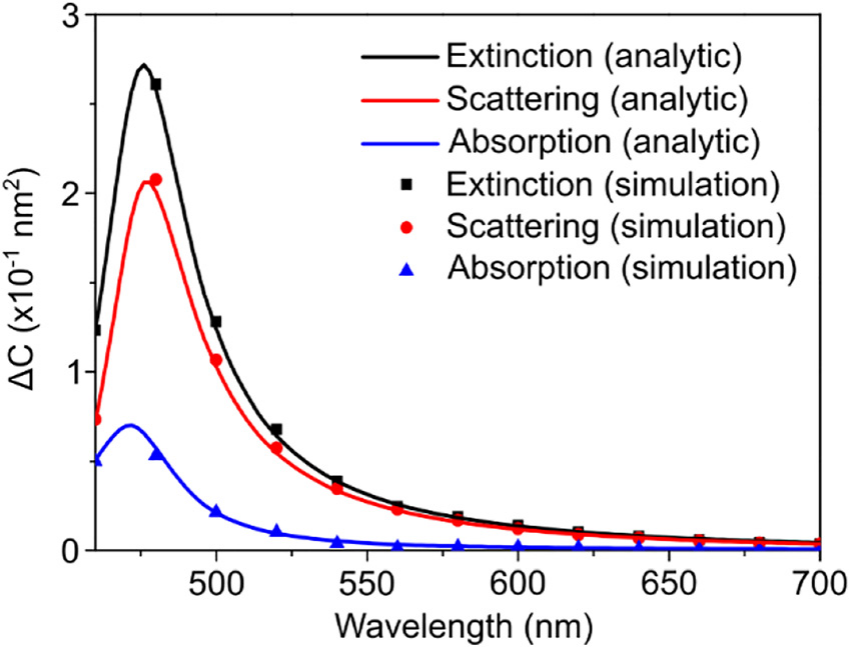
The circular differential cross sections calculated by analytic solution and numerical simulation. The results obtained by the two methods are perfectly matched.

To test the validity of our analytic solutions in Eqs. (8)–(10), we compared the analytic results from (8)–(10) with direct full-field numerical simulations, and present the results in Figure 2. In the electromagnetic simulation, we used a silicon core particle of radius 50 nm and a chiral molecule shell 5 nm thick. The material parameters for silver and silicon are chosen from the tabulated data available in the literature [26, 27]. Optical constants and the chirality parameter of the chiral molecule shell are modeled using the Condon model [28] in accordance with the behavior of typical chiral molecules in the ultraviolet-visible wavelength range [29] (see SI). We chose an aqueous background of permittivity $\epsilon_{b}=1.33^{2}$ . By implementing all the constitutive relations of the chiral medium parameters and other material parameters in COMSOL, we calculated $\Delta C_{ext}^{NP@mol}$ , $\Delta C_{sca}^{NP@mol}$ , and $\Delta C_{abs}^{NP@mol}$ numerically. Figure 2 indicates that the results from the chiral Mie theory agree nicely with the electromagnetic simulation, thereby confirming the validity of the analytic solutions.

## 3 CD of plasmonic and dielectric nanoparticles capped with chiral molecules

Equipped with these analytic tools, we are now able to uncover the role of nanoparticles in CD enhancement. The extinction cross section in Eq. (5) and the circular differential extinction cross section in Eq. (8) allow a quantitative description of enhanced molecular CD by nanoparticle resonances, such as electric/magnetic dipole (*n* = 1), quadrupole (*n* = 2), and octupole (*n* = 3) resonances. The scattering coefficients $\alpha_{n}$ , $\beta_{n}$ , $\gamma_{n}$ and $\delta_{n}$ reflect the chirality parameter κ of the chiral shell as they are determined through the boundary matching conditions. Note that the complex value chirality parameter κ contains the information on the optical rotatory dispersion (ORD) and CD simultaneously. Our chiral Mie theory considers both the ORD and the CD of chiral molecules, unlike a previous work [15] which considered only the ORD of a chiral molecule medium.

**Figure 3:**
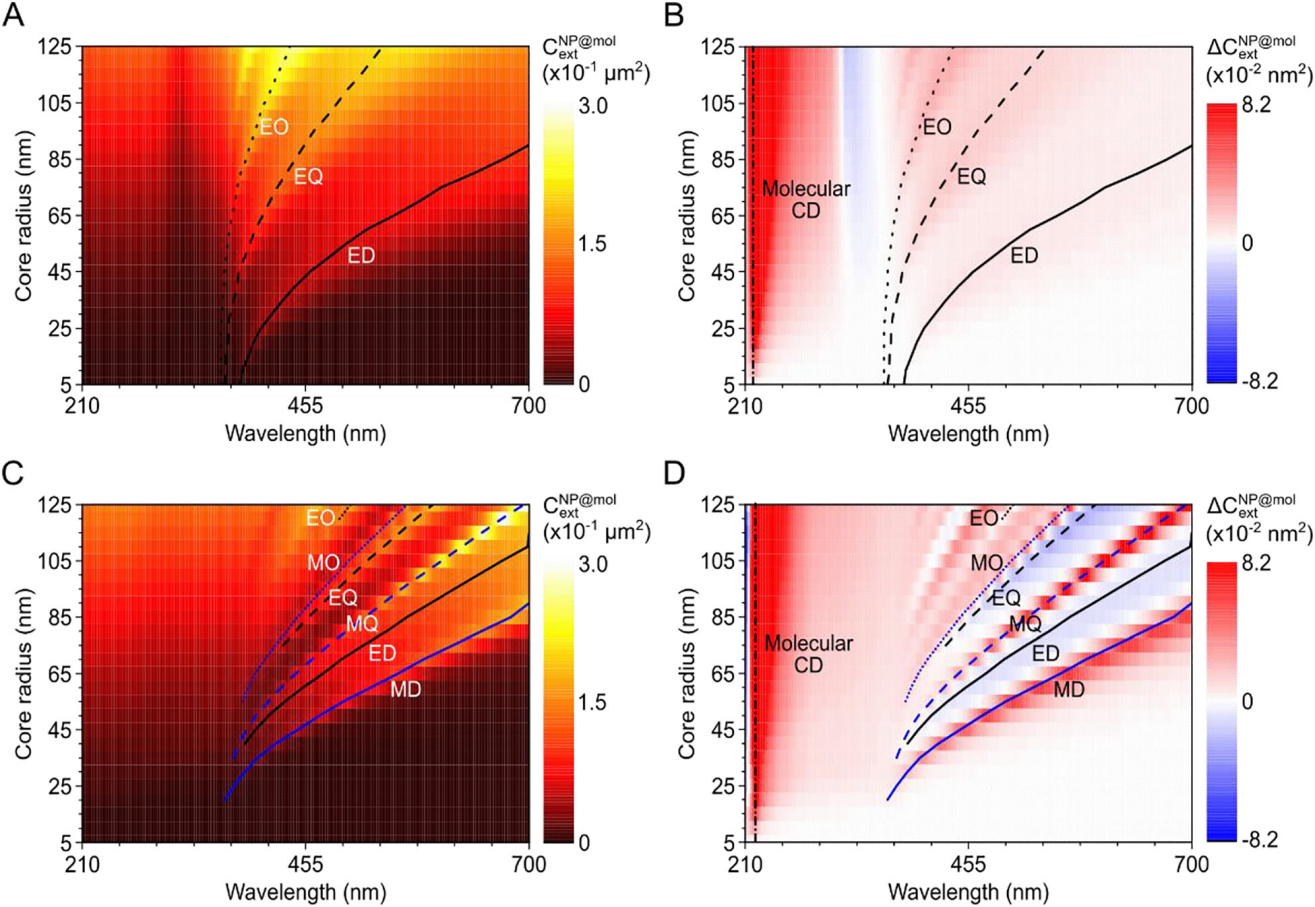
The extinction and circular differential extinction cross section of plasmonic core-chiral shell and dielectric core-chiral shell nanoparticles. (A) The extinction cross section and (B) the circular differential extinction cross section of silver core-chiral shell nanoparticle. (C) The extinction cross section and (D) the circular differential extinction cross section of silicon core-chiral shell nanoparticle. The thickness of the chiral molecular shell is fixed at 1 nm. The ED (black solid), EQ (black dashed), EO (black dotted), MD (blue solid), MQ (blue dashed), and MO (blue dotted) resonance positions are plotted. The black dashed-dotted line shows the molecular intrinsic CD resonance at 220 nm.

Figure 3 shows the extinction cross sections of nanoparticles with a 1 nm thick chiral molecule shell which is the typical thickness of a self-assembled molecular monolayer [30]. The extinction cross section and circular differential extinction cross section of both the silver core case (Figure 3A and B) and the silicon core case (Figure 3C and D) are plotted varying the core size and wavelength. Note that the silver core case shows only electric resonance whereas the silicon core case shows both electric and magnetic resonances. Earlier works focused mostly on the electric dipole resonance and the enhanced CD signal [12], [13], [14], [15] without paying much attention to the higher-order electric resonance modes and induced CD signals. It was considered that dipole resonance plays the dominant role in CD enhancement by nanoparticles and nanostructures, and that complicated higher-order resonances, although present in large-sized particles, have less significant effect on CD enhancement. Contrary to these expectations, our analytic solution reveals the important role of higher-order resonances.

**Figure 4:**
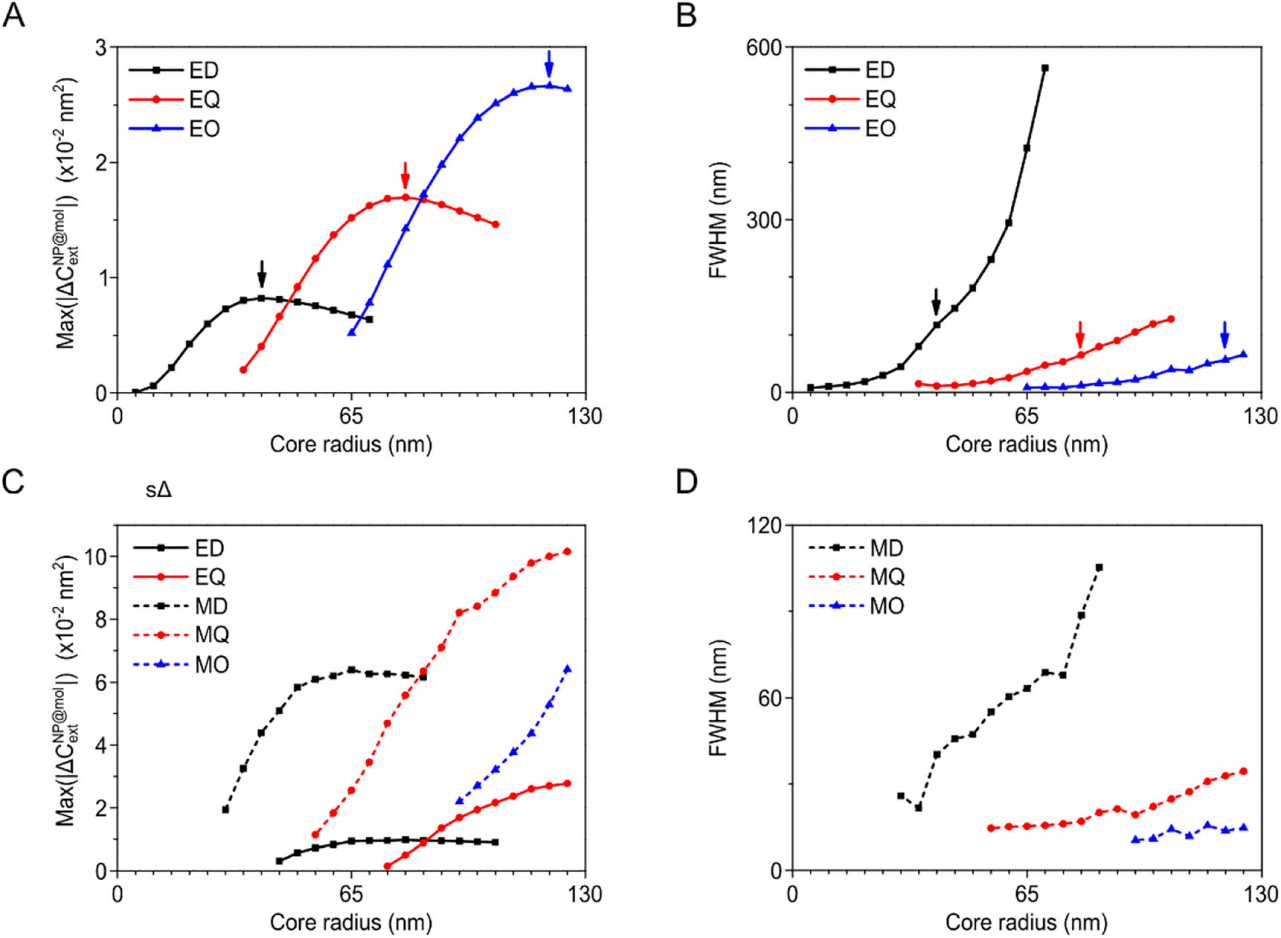
The maximum value of $\left|\Delta C_{ext}^{NP@mol}\right|$ and FWHM of each resonance for varied core radii. (A) And (B) show silver core-chiral shell nanoparticle, (C) and (D) show silicon core-chiral shell nanoparticle. In (A) and (B), the arrow denotes the core radius position at which the $\text{Max}\left(\left|\Delta C_{ext}^{NP@mol}\right|\right)$ is maximized. In Figure 4D, the FWHM for electric resonances are not well defined and thus not plotted.

To clarify the role, from Figure 3, we traced the maximum value of $\left|\Delta C_{ext}^{NP@mol}\right|$ and the full-width half maximum (FWHM) at each resonance for a fixed size of achiral core particle. The results plotted in Figure 4 show the maximum value, $\text{Max}\left(\left|\Delta C_{ext}^{NP@mol}\right|\right)$ , around the electric dipole (ED), the electric quadrupole (EQ) and the electric octupole (EO) resonance, of which the peak values occur at silver core radii of 40 nm (black arrow), 80 nm (red arrow) and 120 nm (blue arrow), respectively. The maximum values of $\text{Max}\left(\left|\Delta C_{ext}^{NP@mol}\right|\right)$ around the EQ and the EO resonance are 2 times and 3.2 times larger, respectively, than the induced CD signal around the ED resonance. Importantly, the FWHM of the induced CD signal in Figure 4B shows that the values at the peak positions of EQ (red arrow) and EO (blue arrow) are 65 nm and 57 nm, respectively, which are smaller than the FWHM value of 117 nm at the ED (black arrow). Thus, we found that, advantageously for CD spectrum measurement, higher-order resonances can provide lager CD magnitudes and smaller CD linewidths than dipole resonance. We point out that the maximal efficiency of ED resonance may be larger than those of higher-order resonances if extinction is normalized by the particle surface area. However, for CD measurement, since the absolute value of a CD signal and the high quality factor of resonance is more important than efficiency, higher-order resonances are more preferable.

The strong CD signal of chiral molecules is usually located in the UV region and the CD signal enhancement by nanoparticle resonances arises in the visible region where molecular CD is much weaker. In fact, the induced CD signal by the ED resonance of a silver nanoparticle is approximately 0.07 times the magnitude of the molecular intrinsic CD signals. However, the situation changes if we consider dielectric core particles. Figure 3C and D show the $C_{ext}^{NP@mol}$ and $\Delta C_{ext}^{NP@mol}$ of the silicon core case, which possesses both electric resonance and magnetic resonance. As noted in the previous circular differential Mie scattering (CDMS) study [15], magnetic resonances show positive differential CD signals while electric resonances show negative ones. Compared to the silver nanoparticle case, the silicon core-chiral shell exhibits much stronger induced CD signals at magnetic resonances, as shown in Figure 4C. For example, the $\text{Max}\left(\left|\Delta C_{ext}^{NP@mol}\right|\right)$ around the MD (MQ) exhibits 6.4 times (7.2 times) larger magnitudes than the ED (EQ) for a silicon core of radius 85 nm. The $\text{Max}\left(\left|\Delta C_{ext}^{NP@mol}\right|\right)$ at the electric resonances of dielectric nanoparticles show similar values to the plasmonic silver nanoparticle case. Figure 4D shows that higher-order magnetic resonances also possess smaller FWHM values compared to those of MD. The FWHM of electric resonances are not well defined and thus not plotted. Thus, we can conclude that the magnetic resonances of high index dielectric nanoparticles induce larger CD signals with smaller CD linewidths than electric resonances or the resonances of plasmonic nanoparticles. All these features are summarized in Table 1.

**Table 1:** Advantages of different multipole resonances for induced CD. X denotes that there are no induced CD signals. The triangle and circle symbols indicate which resonant properties are more advantageous for obtaining the measurable induced CD signals (△ ⟨ ▲ ⟨ ○ ⟨ ●). △ indicates small magnitudes and broad spectral lineshape CD signals. ● indicates large magnitudes and sharp spectral lineshape CD signals.

Core resonances	Core particle		
	Low-index dielectric	Plasmonic (Silver)	High-index dielectric (Silicon)
Electric dipole	X	△	△
Higher-order electric resonances	X	▲	▲
Magnetic dipole	X	X	○
Higher-order magnetic resonances	X	X	●

## 4 Scattering and absorption contribution to CD

Large-sized particles provide an efficient platform for enhancing weak molecular CD signals because they support multipole resonances and a large surface area on which chiral molecules can be adsorbed. When the particle size becomes comparable to the light wavelength, not only the absorption but also the scattering by the particles contributes critically to molecular CD. The CD signals we measure consist of the sum of circular differential scattering and absorption ( $\Delta C_{ext}^{NP@mol}=\Delta C_{sca}^{NP@mol}+\Delta C_{abs}^{NP@mol}$ ). In the previous study, only circular differential absorption was considered for the CD signals of achiral nanostructures coupled with chiral molecules, neglecting scattering [6]. Also, in the CDMS study [15], the scattering contribution was considered only for the spectral change of molecular CD, but not for the intrinsic absorption of the chiral molecular medium. Our chiral Mie theory solution in (5)–(7) allows a qualitative and quantitative understanding of these effects.

**Figure 5:**
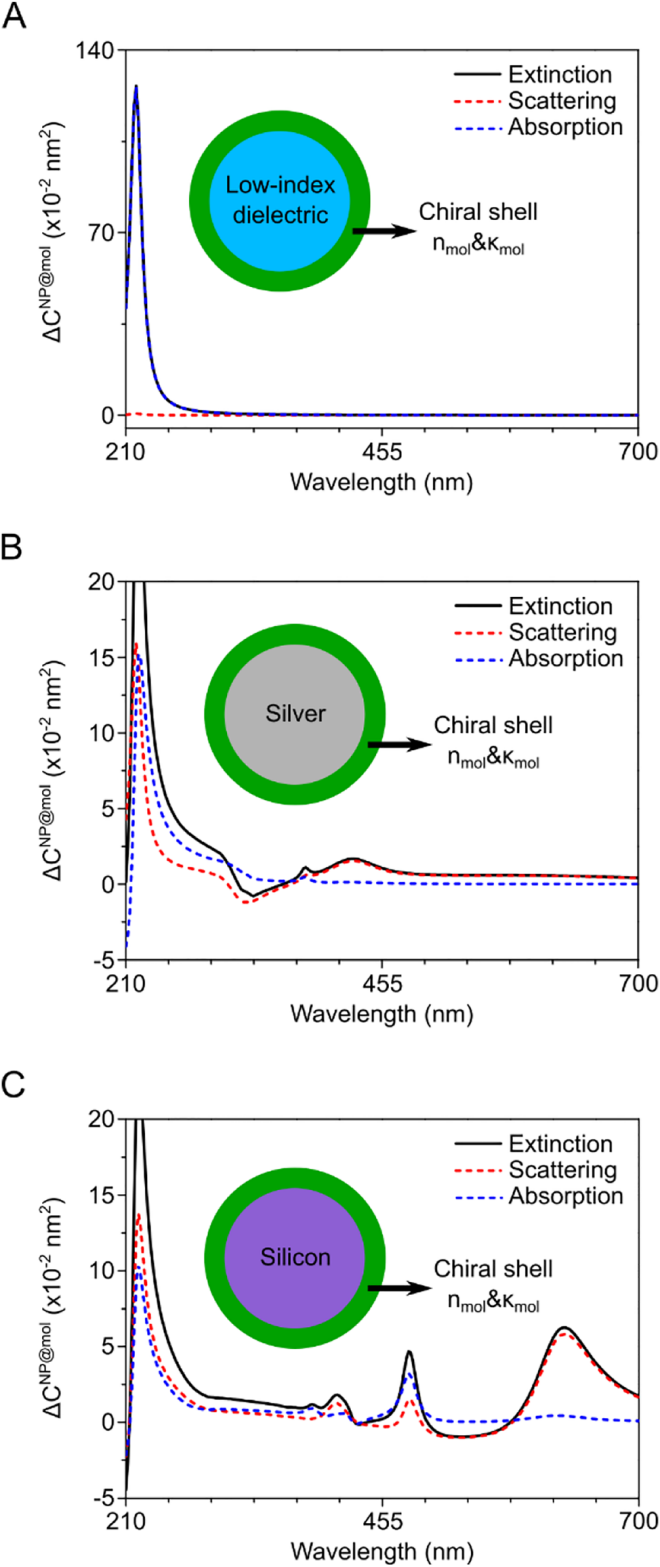
The circular differential extinction (black solid), scattering (red dashed) and absorption (blue dashed) cross section of (A) low-index dielectric core (B) silver core and (C) silicon core particle of radius 75 nm. The refractive index of the low-index dielectric material is the same as the achiral background medium.

Figure 5 shows the effect of particle scattering and absorption on the induced CD. We choose a chiral molecular medium with a CD peak in the UV region to surround a nanoparticle of 75 nm radius and 1 nm shell thickness. Figure 5A shows a low-index (*n* = 1.33) dielectric core particle possessing no resonance and no resulting CD spectral change. In this case, the CD is determined only by the circular differential absorption of the chiral molecular medium and the scattering effect is not noticeable. In the presence of resonance modes, as in Figure 5B (silver core) and C (silicon core), the contribution of scattering to the induced CD cannot be ignored. Particularly, in the present case of the 75 nm particle size, with induced CD at dipole resonances (ED at 428 nm in Figure 5B and MD at 628 nm in Figure 5C), scattering is a dominant factor. This suggests that particle scattering plays a key role in inducing the CD near the multipole resonances as the particle size increases. Thus, it is essential to analyze the scattering properties of nanostructures when designing structures for nanophotonic CD enhancement.

## 5 Conclusions

In this paper, we investigated the effects of the multipole resonances of plasmonic and dielectric nanoparticles on the surrounding chiral molecules. We first developed the chiral Mie theory for an achiral core-chiral shell nanoparticle system and obtained an exact solution for the Mie scattering. Based on the analytic solution, we identified the role of higher-order resonances on CD enhancement. The induced CD signals around the higher-order resonances exhibit larger CD magnitudes with smaller CD linewidths than the dipole resonance case. In particular, we confirmed that high refractive index dielectric nanoparticles supporting magnetic multipoles are more effective for measuring CD signals of adsorbed chiral molecules than the plasmonic nanoparticles that support only electric resonances. In addition, we analyzed the contribution of scattering and absorption to the induced CD signals for large-sized nanoparticles. We showed that, as particle size increases, particle scattering critically contributes to the induced CD near the multipole resonances. In this study, although we only discussed achiral spherical nanoparticles, we believe that our results give insight into non-spherical achiral nanoparticles/nanostructures and that they can be applied to the development of sensitive molecular chirality detection methods.

## Supplementary Material

Supplementary Material
