# Supplementary material for: Molecular chirality detection using plasmonic and dielectric nanoparticles
Source: Nanophotonics. 2022 Jan 11;11(9):1897–904. doi: 10.1515/nanoph-2021-0649 (PMC11502060; doi:10.1515/nanoph-2021-0649)
Supplement: Supplementary file 1 — Supplementary Material [file j_nanoph-2021-0649_suppl.docx]

**Supplementary Information:**

**Molecular chirality detection using plasmonic and dielectric nanoparticles**

TaeHyung Kim^1^ and Q-Han Park^1,*^

^1^Department of Physics, Korea University, Seoul, 02841, Republic of Korea

*E-mail: [qpark@korea.ac.kr](mailto:qpark@korea.ac.kr)

**S1. Derivation of the chiral Mie theory for achiral core-chiral shell nanosphere**

Here, we provide a detailed derivation of the chiral Mie theory describing an achiral core-chiral shell nanosphere embedded in an achiral background medium. We use the wave-field decomposition method to solve the Maxwell’s equation in an isotropic chiral medium  [1]. The electromagnetic constitutive relation for the chiral medium is given by

$$\mathbf{D}=\varepsilon\mathbf{E}+i\kappa\mathbf{H},$$

$$\mathbf{B}=\mu\mathbf{H}-i\kappa\mathbf{E},$$

where $\varepsilon$ is permittivity,$\mu$ is permeability and $\kappa$ is chirality parameter of a chiral medium. Then, the Maxwell’s curl equation in the matrix form is

$$\left(\begin{matrix} \nabla\times\mathbf{E} \\ \nabla\times\mathbf{H} \end{matrix}\right)=\left(\begin{matrix} k_{0}\kappa& ik_{0}\mu\\ -ik_{0}\varepsilon& k_{0}\kappa\end{matrix}\right)\left(\begin{matrix} \mathbf{E} \\ \mathbf{H} \end{matrix}\right),$$

where the vacuum wavenumber $k_{0}=\omega/c$ with the angular frequency $\omega$ and the speed of light $c$. Equation (S3) can be diagonalized as

$$\left(\begin{matrix} \nabla\times\mathbf{Q}_{\mathbf{L}} \\ \nabla\times\mathbf{Q}_{\mathbf{B}} \end{matrix}\right)=\left(\begin{matrix} k_{0}(n+\kappa) & 0 \\ 0 & -k_{0}(n-\kappa) \end{matrix}\right)\left(\begin{matrix} \mathbf{Q}_{\mathbf{L}} \\ \mathbf{Q}_{\mathbf{R}} \end{matrix}\right)=\left(\begin{matrix} k_{L} & 0 \\ 0 & -k_{R} \end{matrix}\right)\left(\begin{matrix} \mathbf{Q}_{\mathbf{L}} \\ \mathbf{Q}_{\mathbf{R}} \end{matrix}\right),$$

with the wavenumber eigenvalues $k_{L}=\left(n+\kappa\right)k_{0}$ and $k_{R}=\left(n-\kappa\right)k_{0}$.

The linear transformation of electromagnetic fields is

$$\left(\begin{matrix} \mathbf{E} \\ \mathbf{H} \end{matrix}\right)=\left(\begin{matrix} 1 & 1 \\ \frac{-i}{\eta} & \frac{i}{\eta} \end{matrix}\right)\left(\begin{matrix} \mathbf{Q}_{\mathbf{L}} \\ \mathbf{Q}_{\mathbf{R}} \end{matrix}\right)=\left(\begin{matrix} 1 & 1 \\ -i\sqrt{\frac{\varepsilon}{\mu}} & i\sqrt{\frac{\varepsilon}{\mu}} \end{matrix}\right)\left(\begin{matrix} \mathbf{Q}_{\mathbf{L}} \\ \mathbf{Q}_{\mathbf{R}} \end{matrix}\right).$$

We utilize the set of wave-field $\left\{\mathbf{Q}_{L},\mathbf{Q}_{R}\right\}$ instead of the set of total electromagnetic fields $\left\{\mathbf{E},\mathbf{H}\right\}$.


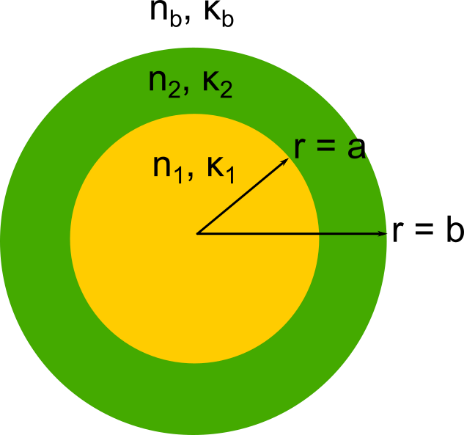
Figure S1. The schematic figure of the chiral Mie theory for a chiral core-chiral shell nanosphere embedded in a chiral medium. The core radius $r=a$ and the radius of entire nanosphere $r=b$.

We firstly consider the chiral core-chiral shell nanosphere embedded in a chiral background medium as shown in the Figure S1. The core particle radius is $a$, the refractive index is $n_{1}$ and the chirality parameter is $\kappa_{1}$. The entire particle radius is $b$. The refractive index and the chirality parameter of shell is $n_{2}$ and $\kappa_{2}$, respectively. The refractive index and the chirality parameter of background medium is $n_{b}$ and $\kappa_{b}$, respectively. The magnetic permeability of nonmagnetic entire system is $\mu_{0}$.

We can write the incident, scattered and internal fields of Figure S1 system in terms of $\left\{\mathbf{Q}_{L},\mathbf{Q}_{R}\right\}$. The incident fields in the background region are expressed as

$$\mathbf{Q}_{L}^{i}=\sum_{n=0}^{\infty} Q_{L}i^{n}\frac{2n+1}{n(n+1)}\left[\left(\mathbf{M}_{o1n}^{(1)}(k_{L})+\mathbf{N}_{o1n}^{(1)}(k_{L})\right)-i\left(\mathbf{M}_{e1n}^{(1)}(k_{L})+\mathbf{N}_{e1n}^{(1)}(k_{L})\right)\right],$$

$$\mathbf{Q}_{R}^{i}=\sum_{n=0}^{\infty} Q_{R}i^{n}\frac{2n+1}{n(n+1)}\left[\left(\mathbf{M}_{o1n}^{(1)}(k_{R})-\mathbf{N}_{o1n}^{(1)}(k_{R})\right)+i\left(\mathbf{M}_{e1n}^{(1)}(k_{R})-\mathbf{N}_{e1n}^{(1)}(k_{R})\right)\right],$$

The polarization of incident fields is controlled by $\left(Q_{L},Q_{R}\right)$: $\left(Q_{L},Q_{R}\right)=\left(1,0\right)$ denotes the left-circularly polarized light (LCP) and $\left(Q_{L},Q_{R}\right)=\left(0,1\right)$ denotes the right-circularly polarized light (RCP). The scattered fields in the background region are expressed as

$$\mathbf{Q}_{L}^{s}=\sum_{n=0}^{\infty} Q_{L}i^{n}\frac{2n+1}{n(n+1)}\left[a_{n}\left(\mathbf{M}_{o1n}^{(3)}(k_{L})+\mathbf{N}_{o1n}^{(3)}(k_{L})\right)-ib_{n}\left(\mathbf{M}_{e1n}^{(3)}(k_{L})+\mathbf{N}_{e1n}^{(3)}(k_{L})\right)\right],$$

$$\mathbf{Q}_{R}^{s}=\sum_{n=0}^{\infty} Q_{R}i^{n}\frac{2n+1}{n(n+1)}\left[c_{n}\left(\mathbf{M}_{o1n}^{(3)}(k_{R})-\mathbf{N}_{o1n}^{(3)}(k_{R})\right)+id_{n}\left(\mathbf{M}_{e1n}^{(3)}(k_{R})-\mathbf{N}_{e1n}^{(3)}(k_{R})\right)\right],$$

The internal fields in core region ($0\leq r\leq a$) are

$$\mathbf{Q}_{L1}=\sum_{n=0}^{\infty} Q_{L}i^{n}\frac{2n+1}{n(n+1)}\left[\alpha_{on}\left(\mathbf{M}_{o1n}^{(1)}(k_{L1})+\mathbf{N}_{o1n}^{(1)}(k_{L1})\right)-i\alpha_{en}\left(\mathbf{M}_{e1n}^{(1)}(k_{L1})+\mathbf{N}_{e1n}^{(1)}(k_{L1})\right)\right],$$

$$\mathbf{Q}_{R1}=\sum_{n=0}^{\infty} Q_{R}i^{n}\frac{2n+1}{n(n+1)}\left[\beta_{on}\left(\mathbf{M}_{o1n}^{(1)}(k_{R1})-\mathbf{N}_{o1n}^{(1)}(k_{R1})\right)+i\beta_{en}\left(\mathbf{M}_{e1n}^{(1)}(k_{R1})-\mathbf{N}_{e1n}^{(1)}(k_{R1})\right)\right],$$

and the internal fields in the shell region ($a<r\leq b$) are

$$\mathbf{Q}_{L2}=\sum_{n=0}^{\infty} Q_{L}i^{n}\frac{2n+1}{n(n+1)}\left[\begin{matrix} f_{on}\left(\mathbf{M}_{o1n}^{(1)}(k_{L2})+\mathbf{N}_{o1n}^{(1)}(k_{L2})\right)-if_{en}\left(\mathbf{M}_{e1n}^{(1)}(k_{L2})+\mathbf{N}_{e1n}^{(1)}(k_{L2})\right) \\ +g_{on}\left(\mathbf{M}_{o1n}^{(2)}(k_{L2})+\mathbf{N}_{o1n}^{(2)}(k_{L2})\right)-ig_{en}\left(\mathbf{M}_{e1n}^{(2)}(k_{L2})+\mathbf{N}_{e1n}^{(2)}(k_{L2})\right) \end{matrix}\right],$$

$$\mathbf{Q}_{R2}=\sum_{n=0}^{\infty} Q_{R}i^{n}\frac{2n+1}{n(n+1)}\left[\begin{matrix} v_{on}\left(\mathbf{M}_{o1n}^{(1)}(k_{R2})-\mathbf{N}_{o1n}^{(1)}(k_{R2})\right)+iv_{en}\left(\mathbf{M}_{e1n}^{(1)}(k_{R2})-\mathbf{N}_{e1n}^{(1)}(k_{R2})\right) \\ +w_{on}\left(\mathbf{M}_{o1n}^{(2)}(k_{R2})-\mathbf{N}_{o1n}^{(2)}(k_{R2})\right)+iw_{en}\left(\mathbf{M}_{e1n}^{(2)}(k_{R2})-\mathbf{N}_{e1n}^{(2)}(k_{R2})\right) \end{matrix}\right],$$

The wavenumber $k_{L1,R1}=\left(n_{1}\pm\kappa_{1}\right)k_{0}$ in the core region, $k_{L2,R2}=\left(n_{2}\pm\kappa_{2}\right)k_{0}$ in the shell region and $k_{L,R}=\left(n_{b}\pm\kappa_{b}\right)k_{0}$ in the background. The expansion coefficients of scattered fields are $a_{n}$, $b_{n}$, $c_{n}$ and $d_{n}$. The expansion coefficients of internals fields are $\alpha_{on}$, $\alpha_{en}$, $\beta_{on}$, $\beta_{en}$, $f_{on}$, $f_{en}$, $g_{on}$, $g_{en}$, $v_{on}$, $v_{en}$, $w_{on}$ while $w_{en}$. $\mathbf{M}_{omn}$, $\mathbf{M}_{emn}$, $\mathbf{N}_{omn}$ and $\mathbf{N}_{emn}$ are the vector spherical harmonics where subscripts $e$ and $o$ denote even and odd. The order of vector spherical harmonics $m$ is fixed to 1 [1]. The vector spherical harmonics are given by

where superscripts of $\mathbf{M}$ and $\mathbf{N}$are suppressed and$z_{n}$stands for the spherical Bessel function. If the superscript is (1), $z_{n}$is the spherical Bessel function $j_{n}$, for (2) $z_{n}$is the spherical Neumann function $y_{n}$, for (3)$z_{n}$is the spherical Hankel functions of first kind $h_{n}^{\left(1\right)}$. $\pi_{n}=\frac{P_{n}^{1}}{\sin\theta}$ and $\tau_{n}=\frac{dP_{n}^{1}}{d\theta}$ with the associated Legendre Polynomials $P_{n}^{m}$ [1].

The expansion coefficients of scattered and internal fields are determined by boundary conditions. At the surface of the core particle ($r=a$), the boundary condition is

$$\left(\mathbf{E}_{2}-\mathbf{E}_{1}\right)\times\hat{\mathbf{e}}_{r}=0,$$

$$\left(\mathbf{H}_{2}-\mathbf{H}_{1}\right)\times\hat{\mathbf{e}}_{r}=0.$$

At the outer surface of a shell ($r=b$), the boundary condition is

$$\left(\mathbf{E}_{s}+\mathbf{E}_{i}-\mathbf{E}_{2}\right)\times\hat{\mathbf{e}}_{r}=0,$$

$$\left(\mathbf{H}_{s}+\mathbf{H}_{i}-\mathbf{H}_{2}\right)\times\hat{\mathbf{e}}_{r}=0,$$

where $\hat{\mathbf{e}}_{r}$ is the radial unit vector. Using boundary conditions, we obtain 16 equations for the expansion coefficients. These equations can be expressed in the following matrix form;

$$\mathbf{M}_{1}\left(\begin{matrix} \alpha_{en} \\ \vdots\\ w_{on} \end{matrix}\right)=\mathbf{M}_{2},$$

where $\left(\begin{matrix} \alpha_{en} \\ \vdots\\ w_{on} \end{matrix}\right)$ contains 16 expansion coefficients in the following order: ($\alpha_{en}$, $\beta_{en}$, $\alpha_{on}$, $\beta_{on}$, $b_{n}$, $d_{n}$, $a_{n}$, $c_{n}$, $f_{en}$, $g_{en}$, $v_{en}$, $w_{en}$, $f_{on}$, $g_{on}$, $v_{on}$, $w_{on}$).

The matrix $\mathbf{M}_{1}$ (16 x 16 matrix) is written as

$$\mathbf{M}_{1}=\left(\begin{matrix} \mathbf{A} & \mathbf{0} & \mathbf{B} & \mathbf{0} \\ \mathbf{C} & \mathbf{0} & \mathbf{0} & \mathbf{D} \\ \mathbf{0} & \mathbf{E} & \mathbf{F} & \mathbf{0} \\ \mathbf{0} & \mathbf{G} & \mathbf{0} & \mathbf{H} \end{matrix}\right),$$

The $\mathbf{A}$, $\mathbf{B}$, $\mathbf{C}$, $\mathbf{D}$, $\mathbf{E}$, $\mathbf{F}$, $\mathbf{G}$, $\mathbf{H}$ and $\mathbf{0}$ are 4 x 4 matrix.

$$\mathbf{0}=\left(\begin{matrix} 0 & 0 & 0 & 0 \\ 0 & 0 & 0 & 0 \\ 0 & 0 & 0 & 0 \\ 0 & 0 & 0 & 0 \end{matrix}\right),$$

$$\mathbf{A}=\left(\begin{matrix} Q_{L}j_{n}(k_{L1}a) & -Q_{R}j_{n}(k_{R1}a) & 0 & 0 \\ Q_{L}\frac{[k_{L1}aj_{n}(k_{L1}a)]'}{k_{L1}a} & Q_{R}\frac{[k_{R1}aj_{n}(k_{R1}a)]'}{k_{R1}a} & 0 & 0 \\ -\frac{m_{1}}{m_{2}}Q_{L}j_{n}(k_{L1}a) & -\frac{m_{1}}{m_{2}}Q_{R}j_{n}(k_{R1}a) & 0 & 0 \\ -\frac{m_{1}}{m_{2}}Q_{L}\frac{[k_{L1}aj_{n}(k_{L1}a)]'}{k_{L1}a} & \frac{m_{1}}{m_{2}}Q_{R}\frac{[k_{R1}aj_{n}(k_{R1}a)]'}{k_{R1}a} & 0 & 0 \end{matrix}\right),$$

$$\mathbf{B}=\left(\begin{matrix} -Q_{L}j_{n}(k_{L2}a) & -Q_{L}y_{n}(k_{L2}a) & Q_{R}j_{n}(k_{R2}a) & Q_{R}y_{n}(k_{R2}a) \\ -Q_{L}\frac{[k_{L2}aj_{n}(k_{L2}a)]'}{k_{L2}a} & -Q_{L}\frac{[k_{L2}ay_{n}(k_{L2}a)]'}{k_{L2}a} & -Q_{R}\frac{[k_{R2}aj_{n}(k_{R2}a)]'}{k_{R2}a} & -Q_{R}\frac{[k_{R2}ay_{n}(k_{R2}a)]'}{k_{R2}a} \\ Q_{L}j_{n}(k_{L2}a) & Q_{L}y_{n}(k_{L2}a) & Q_{R}j_{n}(k_{R2}a) & Q_{R}y_{n}(k_{R2}a) \\ Q_{L}\frac{[k_{L2}aj_{n}(k_{L2}a)]'}{k_{L2}a} & Q_{L}\frac{[k_{L2}ay_{n}(k_{L2}a)]'}{k_{L2}a} & -Q_{R}\frac{[k_{R2}aj_{n}(k_{R2}a)]'}{k_{R2}a} & -Q_{R}\frac{[k_{R2}ay_{n}(k_{R2}a)]'}{k_{R2}a} \end{matrix}\right),$$

$$\mathbf{C}=\left(\begin{matrix} 0 & 0 & Q_{L}j_{n}(k_{L1}a) & Q_{R}j_{n}(k_{R1}a) \\ 0 & 0 & Q_{L}\frac{[k_{L1}aj_{n}(k_{L1}a)]'}{k_{L1}a} & -Q_{R}\frac{[k_{R1}aj_{n}(k_{R1}a)]'}{k_{R1}a} \\ 0 & 0 & -\frac{m_{1}}{m_{2}}Q_{L}j_{n}(k_{L1}a) & \frac{m_{1}}{m_{2}}Q_{R}j_{n}(k_{R1}a) \\ 0 & 0 & -\frac{m_{1}}{m_{2}}Q_{L}\frac{[k_{L1}aj_{n}(k_{L1}a)]'}{k_{L1}a} & -\frac{m_{1}}{m_{2}}Q_{R}\frac{[k_{R1}aj_{n}(k_{R1}a)]'}{k_{R1}a} \end{matrix}\right),$$

$$\mathbf{D}=\left(\begin{matrix} -Q_{L}j_{n}(k_{L2}a) & -Q_{L}y_{n}(k_{L2}a) & -Q_{R}j_{n}(k_{R2}a) & -Q_{R}y_{n}(k_{R2}a) \\ -Q_{L}\frac{[k_{L2}aj_{n}(k_{L2}a)]'}{k_{L2}a} & -Q_{L}\frac{[k_{L2}ay_{n}(k_{L2}a)]'}{k_{L2}a} & Q_{R}\frac{[k_{R2}aj_{n}(k_{R2}a)]'}{k_{R2}a} & Q_{R}\frac{[k_{R2}ay_{n}(k_{R2}a)]'}{k_{R2}a} \\ Q_{L}j_{n}(k_{L2}a) & Q_{L}y_{n}(k_{L2}a) & -Q_{R}j_{n}(k_{R2}a) & -Q_{R}y_{n}(k_{R2}a) \\ Q_{L}\frac{[k_{L2}aj_{n}(k_{L2}a)]'}{k_{L2}a} & Q_{L}\frac{[k_{L2}ay_{n}(k_{L2}a)]'}{k_{L2}a} & Q_{R}\frac{[k_{R2}aj_{n}(k_{R2}a)]'}{k_{R2}a} & Q_{R}\frac{[k_{R2}ay_{n}(k_{R2}a)]'}{k_{R2}a} \end{matrix}\right),$$

$$\mathbf{E}=\left(\begin{matrix} Q_{L}h_{n}^{(1)}(k_{L}b) & -Q_{R}h_{n}^{(1)}(k_{R}b) & 0 & 0 \\ Q_{L}\frac{[k_{L}bh_{n}^{(1)}(k_{L}b)]'}{k_{L}b} & Q_{R}\frac{[k_{R}bh_{n}^{(1)}(k_{R}b)]'}{k_{R}b} & 0 & 0 \\ -Q_{L}h_{n}^{(1)}(k_{L}b) & -Q_{R}h_{n}^{(1)}(k_{R}b) & 0 & 0 \\ -Q_{L}\frac{[k_{L}bh_{n}^{(1)}(k_{L}b)]'}{k_{L}b} & Q_{R}\frac{[k_{R}bh_{n}^{(1)}(k_{R}b)]'}{k_{R}b} & 0 & 0 \end{matrix}\right),$$

$$\mathbf{F}=\left(\begin{matrix} -Q_{L}j_{n}(k_{L2}b) & -Q_{L}y_{n}(k_{L2}b) & Q_{R}j_{n}(k_{R2}b) & Q_{R}y_{n}(k_{R2}b) \\ -Q_{L}\frac{[k_{L2}bj_{n}(k_{L2}b)]'}{k_{L2}b} & -Q_{L}\frac{[k_{L2}by_{n}(k_{L2}b)]'}{k_{L2}b} & -Q_{R}\frac{[k_{R2}bj_{n}(k_{R2}b)]'}{k_{R2}b} & -Q_{R}\frac{[k_{R2}by_{n}(k_{R2}b)]'}{k_{R2}b} \\ m_{2}Q_{L}j_{n}(k_{L2}b) & m_{2}Q_{L}y_{n}(k_{L2}b) & m_{2}Q_{R}j_{n}(k_{R2}b) & m_{2}Q_{R}y_{n}(k_{R2}b) \\ m_{2}Q_{L}\frac{[k_{L2}bj_{n}(k_{L2}b)]'}{k_{L2}b} & m_{2}Q_{L}\frac{[k_{L2}by_{n}(k_{L2}b)]'}{k_{L2}b} & -m_{2}Q_{R}\frac{[k_{R2}bj_{n}(k_{R2}b)]'}{k_{R2}b} & -m_{2}Q_{R}\frac{[k_{R2}by_{n}(k_{R2}b)]'}{k_{R2}b} \end{matrix}\right),$$

$$\mathbf{G}=\left(\begin{matrix} 0 & 0 & Q_{L}h_{n}^{(1)}(k_{L}b) & Q_{R}h_{n}^{(1)}(k_{R}b) \\ 0 & 0 & Q_{L}\frac{[k_{L}bh_{n}^{(1)}(k_{L}b)]'}{k_{L}b} & -Q_{R}\frac{[k_{R}bh_{n}^{(1)}(k_{R}b)]'}{k_{R}b} \\ 0 & 0 & -Q_{L}h_{n}^{(1)}(k_{L}b) & Q_{R}h_{n}^{(1)}(k_{R}b) \\ 0 & 0 & -Q_{L}\frac{[k_{L}bh_{n}^{(1)}(k_{L}b)]'}{k_{L}b} & -Q_{R}\frac{[k_{R}bh_{n}^{(1)}(k_{R}b)]'}{k_{R}b} \end{matrix}\right),$$

$$\mathbf{H}=\left(\begin{matrix} -Q_{L}j_{n}(k_{L2}b) & -Q_{L}y_{n}(k_{L2}b) & -Q_{R}j_{n}(k_{R2}b) & -Q_{R}y_{n}(k_{R2}b) \\ -Q_{L}\frac{[k_{L2}bj_{n}(k_{L2}b)]'}{k_{L2}b} & -Q_{L}\frac{[k_{L2}by_{n}(k_{L2}b)]'}{k_{L2}b} & Q_{R}\frac{[k_{R2}bj_{n}(k_{R2}b)]'}{k_{R2}b} & Q_{R}\frac{[k_{R2}by_{n}(k_{R2}b)]'}{k_{R2}b} \\ m_{2}Q_{L}j_{n}(k_{L2}b) & m_{2}Q_{L}y_{n}(k_{L2}b) & -m_{2}Q_{R}j_{n}(k_{R2}b) & -m_{2}Q_{R}y_{n}(k_{R2}b) \\ m_{2}Q_{L}\frac{[k_{L2}bj_{n}(k_{L2}b)]'}{k_{L2}b} & m_{2}Q_{L}\frac{[k_{L2}by_{n}(k_{L2}b)]'}{k_{L2}b} & m_{2}Q_{R}\frac{[k_{R2}bj_{n}(k_{R2}b)]'}{k_{R2}b} & m_{2}Q_{R}\frac{[k_{R2}by_{n}(k_{R2}b)]'}{k_{R2}b} \end{matrix}\right)$$

The matrix $\mathbf{M}_{2}$ (16 x 1 matrix) is written as

$$\mathbf{M}_{2}=\left(\begin{matrix} 0 \\ 0 \\ 0 \\ 0 \\ 0 \\ 0 \\ 0 \\ 0 \\ -Q_{L}j_{n}(k_{L}b)+Q_{R}j_{n}(k_{R}b) \\ -Q_{L}\frac{[k_{L}bj_{n}(k_{L}b)]'}{k_{L}b}-Q_{R}\frac{[k_{R}bj_{n}(k_{R}b)]'}{k_{R}b} \\ Q_{L}j_{n}(k_{L}b)+Q_{R}j_{n}(k_{R}b) \\ Q_{L}\frac{[k_{L}bj_{n}(k_{L}b)]'}{k_{L}b}-Q_{R}\frac{[k_{R}bj_{n}(k_{R}b)]'}{k_{R}b} \\ -Q_{L}j_{n}(k_{L}b)-Q_{R}j_{n}(k_{R}b) \\ -Q_{L}\frac{[k_{L}bj_{n}(k_{L}b)]'}{k_{L}b}+Q_{R}\frac{[k_{R}bj_{n}(k_{R}b)]'}{k_{R}b} \\ Q_{L}j_{n}(k_{L}b)-Q_{R}j_{n}(k_{R}b) \\ Q_{L}\frac{[k_{L}bj_{n}(k_{L}b)]'}{k_{L}b}+Q_{R}\frac{[k_{R}bj_{n}(k_{R}b)]'}{k_{R}b} \end{matrix}\right),$$

where the relative refractive index $m_{1}=k_{core}/k_{b}$ and $m_{2}=k_{shell}/k_{b}$. The wavenumber $k_{core}=n_{1}k_{0}$ in the core region, the wavenumber $k_{shell}=n_{2}k_{0}$ in the shell region and the wavenumber $k_{b}=n_{b}k_{0}$ in the background.

When the chirality parameters of core region and the background region are turned off ($\kappa_{1}=\kappa_{b}=0$), we can obtain the solution for the achiral core-chiral shell nanosphere embedded in an achiral background medium. Solving equation (S20) in this situation, the expansion coefficients of scattered fields are arranged in the following form

$$a_{n}=\frac{1}{Q_{L}}\left(Q_{L}\alpha_{n}+Q_{R}\beta_{n}\right),$$

$$b_{n}=\frac{1}{Q_{L}}\left(Q_{L}\alpha_{n}-Q_{R}\beta_{n}\right),$$

$$c_{n}=\frac{1}{Q_{R}}\left(Q_{L}\gamma_{n}+Q_{R}\delta_{n}\right),$$

$$d_{n}=\frac{1}{Q_{R}}\left(-Q_{L}\gamma_{n}+Q_{R}\delta_{n}\right),$$

The $\alpha_{n}$, $\beta_{n}$, $\gamma_{n}$ and $\delta_{n}$ are scattering coefficients that carry the chirality of the shell region $\kappa_{2}$.

The extinction and scattered energy rates are given by [1]

The intensity of incident fields is $I_{i}=\left(Q_{L}^{2}+Q_{R}^{2}\right)/\eta$.

Consequently, we obtain the extinction, scattering and absorption cross section

$$C_{ext}=\frac{W_{ext}}{I_{i}}=-4\pi\sum_{n=1}^{\infty} \left(2n+1\right)\left[\frac{Q_{L}^{2}}{Q_{L}^{2}+Q_{R}^{2}}\frac{\mathrm{Re}\alpha_{n}}{k_{b}^{2}}+\frac{Q_{R}^{2}}{Q_{L}^{2}+Q_{R}^{2}}\frac{\mathrm{Re}\delta_{n}}{k_{b}^{2}}\right],$$

$$C_{sca}=\frac{W_{sca}}{I_{i}}=4\pi\sum_{n=1}^{\infty} \left(2n+1\right)\left[\frac{Q_{L}^{2}}{Q_{L}^{2}+Q_{R}^{2}}\frac{\left|\alpha_{n}\right|^{2}+\left|\gamma_{n}\right|^{2}}{k_{b}^{2}}+\frac{Q_{R}^{2}}{Q_{L}^{2}+Q_{R}^{2}}\frac{\left|\beta_{n}\right|^{2}+\left|\delta_{n}\right|^{2}}{k_{b}^{2}}\right],$$

$$C_{abs}=C_{ext}-C_{abs}$$

and the circular differential extinction, scattering and absorption is

$$\begin{matrix} \Delta C_{ext}=C_{ext}\left(Q_{L}=1,Q_{R}=0\right)-C_{ext}\left(Q_{L}=0,Q_{R}=1\right) \\ =-4\pi\sum_{n=1}^{\infty} \left(2n+1\right)\left[\frac{\mathrm{Re}\alpha_{n}-\mathrm{Re}\delta_{n}}{k_{b}^{2}}\right], \end{matrix}$$

$$\begin{matrix} \Delta C_{sca}=C_{sca}\left(Q_{L}=1,Q_{R}=0\right)-C_{sca}\left(Q_{L}=0,Q_{R}=1\right) \\ =4\pi\sum_{n=1}^{\infty} \left(2n+1\right)\left[\frac{\left|\alpha_{n}\right|^{2}-\left|\beta_{n}\right|^{2}+\left|\gamma_{n}\right|^{2}-\left|\delta_{n}\right|^{2}}{k_{b}^{2}}\right], \end{matrix}$$

$$\Delta C_{abs}=C_{abs}\left(Q_{L}=1,Q_{R}=0\right)-C_{abs}\left(Q_{L}=0,Q_{R}=1\right).$$

**S2. Optical properties of a molecular chiral medium**

The dispersive permittivity and the chirality parameter of chiral medium are expressed by

$$\varepsilon_{mol}=\varepsilon_{solv}+Nd\omega_{0}\left(\frac{\omega_{0}^{2}-\omega^{2}}{\left(\omega_{0}^{2}-\omega^{2}\right)^{2}+\omega^{2}\Gamma^{2}}+i\frac{\omega\Gamma}{\left(\omega_{0}^{2}-\omega^{2}\right)^{2}+\omega^{2}\Gamma^{2}}\right)$$

$$\kappa_{mol}=Nr\omega\left(\frac{\omega_{0}^{2}-\omega^{2}}{\left(\omega_{0}^{2}-\omega^{2}\right)^{2}+\omega^{2}\Gamma^{2}}+i\frac{\omega\Gamma}{\left(\omega_{0}^{2}-\omega^{2}\right)^{2}+\omega^{2}\Gamma^{2}}\right)$$

with the relative permittivity of a solvent $\varepsilon_{solv}$, the molecular number density $N$, the electric dipole strength $d$, the molecular rotatory strength $r$, the absorption band frequency $\omega_{0}$ and the damping factor $\Gamma$. We set the parameter so that the dissymmetry factor $g$ of chiral molecular medium has the value of $10^{-3}\sim10^{-4}$ order in the ultraviolet-visible wavelength range [2]. The values of the parameters are $\varepsilon_{solv}=1.33^{2}$, $Nd=0.11\times10^{15}\text{ s}^{-1}$, $Nr=0.81\times10^{11}\text{ s}^{-1}$, $\omega_{0}=2\pi c/(220\text{ nm)}$ and $\Gamma=5.2\times10^{14}\text{ s}^{-1}$. Figure S3 shows the permittivity, the chirality parameter, and the dissymmetric factor of chiral molecular medium with path length 1 mm.


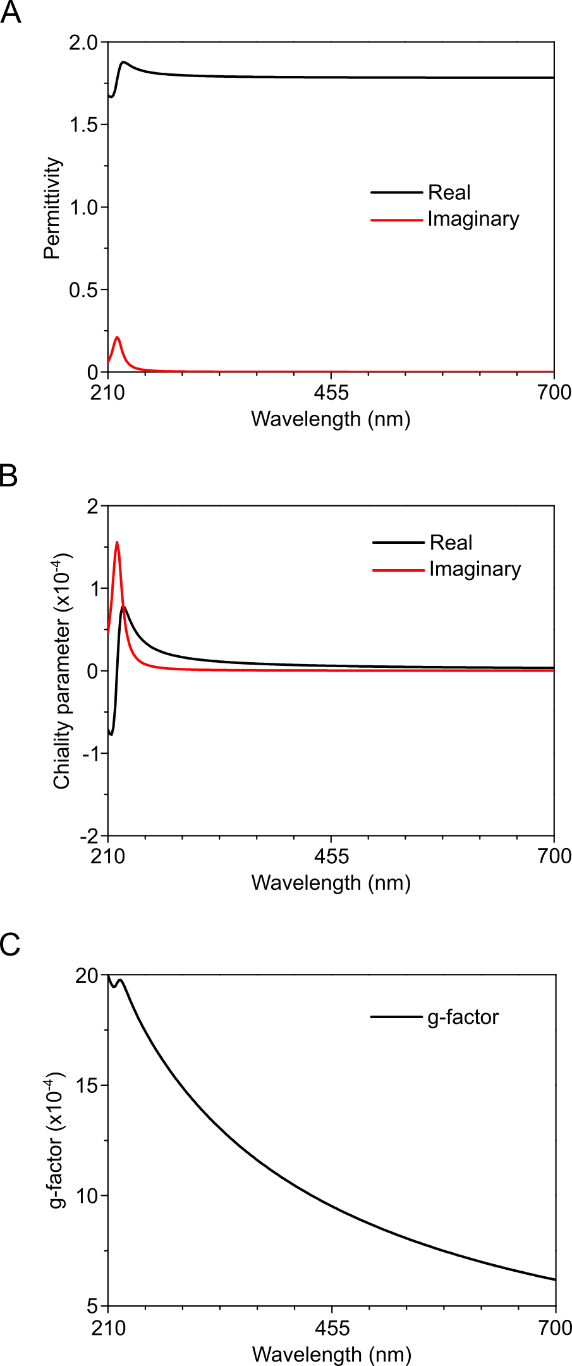


Figure S3. (A) the permittivity (B) the chirality parameter and (C) g-factor of chiral molecular medium. The properties are modeled by the Condon model. The path length of chiral molecular medium is 1 mm.

**S4. Reference**

[1] Bohren CF, Huffman DR. Absorption and Scattering of Light by Small Particles. New Jersey, United States, John Wiley & Sons, Inc., 1998.

[2] Inoue Y, Ramamurthy V. Chiral Photochemistry, New York, United States, Marcel Dekker, 2004.
